# Invasive lobular carcinoma of the breast with colonic metastasis: a case series of three patients

**DOI:** 10.1186/s40792-023-01762-0

**Published:** 2023-10-17

**Authors:** Shinya Otsuka, Kazuteru Komuro, Masato Suzuoki, Shuhei Hayasaka, Momoko Tsuda, Kimitoshi Kubo, Tomone Ueki, Yumi Moriya, Noriko Kimura, Masanori Ohara

**Affiliations:** 1 Department of Surgery, National Hospital Organization (NHO) Hakodate National Hospital, 18-16, Kawahara-Cho, Hakodate, Hokkaido 041-8512 Japan; 2 Department of Gastroenterology, NHO Hakodate National Hospital, Hakodate, Hokkaido Japan; 3 Department of Diagnostic Pathology, NHO Hakodate National Hospital, Hakodate, Hokkaido Japan

**Keywords:** Invasive lobular carcinoma, Breast cancer, Colonic metastasis, Colonoscopy, Surgical intervention, Systemic treatment

## Abstract

**Background:**

Although metastatic spread of breast cancer to the gastrointestinal tract is very rare, it is more likely to occur in invasive lobular carcinoma (ILC) than in ductal carcinoma. Colonic metastasis is particularly rare, and the treatment strategies for these cases are not clearly defined. Herein, we report three cases of ILC with various abdominal symptoms associated with colonic metastasis.

**Case presentation:**

*Case 1* A 70-year-old female patient with vomiting and melena was referred to our hospital. Endoscopic examination revealed a Dieulafoy ulcer in the rectum and an elevated lesion in the descending colon. She also had two breast nodules, and was diagnosed as ILC with colonic metastasis. Considering her general condition, the best supportive care (BSC) was offered. The patient died 4 months after confirmation of the diagnosis. *Case 2* An 80-year-old female patient presented with diarrhea and vomiting. She was diagnosed with ILC with colonic metastasis, and a coloscopy revealed stenosis of the transverse colon with a metastatic lesion. Ileosigmoid bypass surgery was performed for intestinal obstruction, and systemic treatment for breast cancer was initiated. The patient developed peritoneal carcinomatosis and died 1 year and 2 months after surgery. *Case 3* A 56-year-old female patient underwent left total mastectomy for ILC, and laparoscopic transverse colectomy was conducted for a colonic lesion 9 years and 2 months after. The diagnosis as colonic metastasis was not confirmed at that time. Two years and 2 months later, torose lesions were detected in the hepatic flexural and descending colon, and histopathological findings indicated that all colon tumors, including the previously resected tumor, were metastatic spread of ILC. Systemic treatment was continued, but the transverse colonic lesion penetrated the abdominal wall, and an abscess formed 2 years and 11 months after the resection. The fistula improved by continuous suction drainage following ileostomy but recurred, and the patient died 3 years and 8 months after colectomy.

**Conclusions:**

Colonic metastases from breast cancer can trigger various abdominal symptoms, and the prognosis in these cases is generally poor. In selected cases, surgical treatment for abdominal symptoms and subsequent systemic therapy can contribute to a prolonged prognosis.

## Background

Breast cancer is the most prevalent malignancy in women worldwide, with an age-adjusted mortality rate of 13.6% [1]. As it progresses, hematogenous and lymphatic metastases are often observed, and the most commonly involved organs are the liver, lungs, brain, and bones [2]. Spread to the gastrointestinal tract is relatively rare; McLemore et al. reported that it was found in 73 of 12,001 cases of primary breast cancer [3]. In contrast, a recent study reported that 11% of autopsy cases with distant metastases had colonic foci [4]. Invasive lobular carcinoma (ILC) accounts for 10–15% of breast cancers [5] and is more likely to metastasize to the gastrointestinal tract than invasive ductal carcinoma [6, 7]. Colorectal metastasis might be even rarer than involvement of the stomach and small intestine [3, 8]. The clinical presentation of gastrointestinal lesions varies, and the treatment strategies for these cases are not clearly defined. Here, we report three cases of ILC with various abdominal symptoms due to colonic metastases including one previously reported case [9].

## Case presentation

### Case 1

**Fig. 1:**
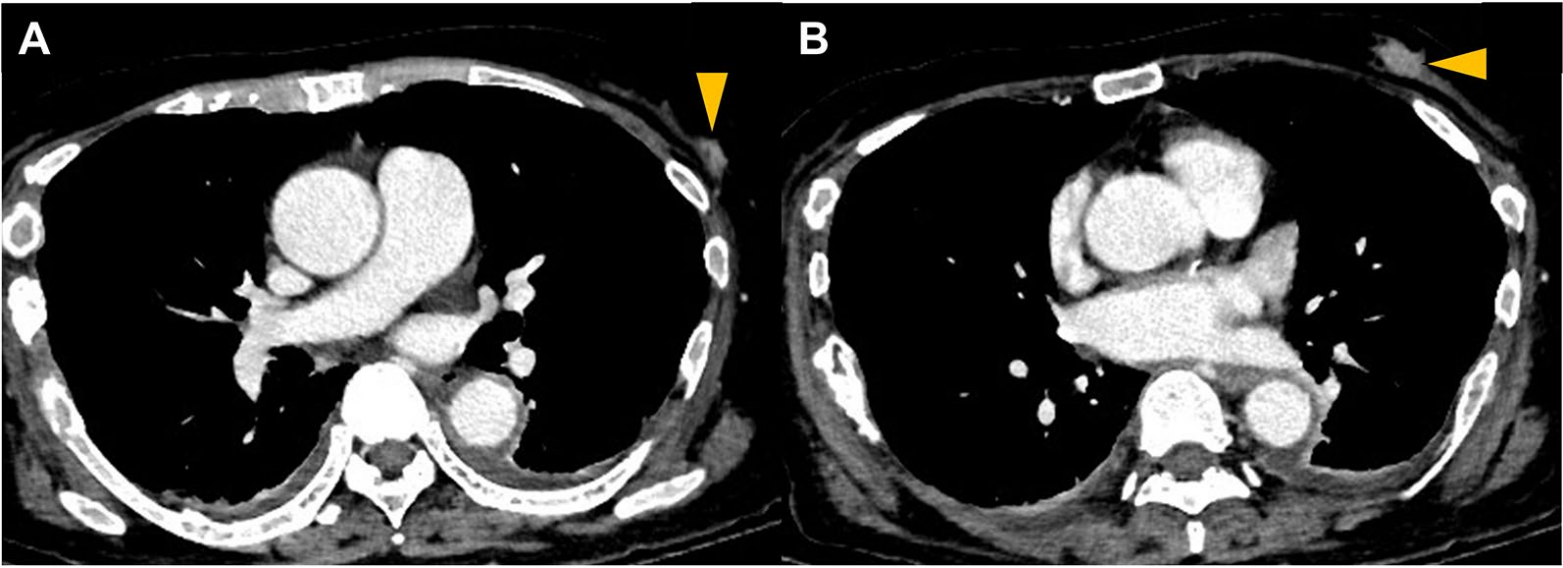
Computed tomography findings of Case 1. Two nodules are visible in the left EAC and C segments of the mammary gland (arrowheads)

A 70-year-old female patient who underwent rehabilitation following a subdural hemorrhage was transferred to the hospital because of repeated vomiting and melena. Computed tomography (CT) scan revealed two nodules in the left mammary gland, multiple osteolytic changes, and fractures throughout the body (Fig. 1). Endoscopic examination suggested bleeding from a Dieulafoy ulcer in the rectum, and electrocoagulation was performed.

**Fig. 2:**
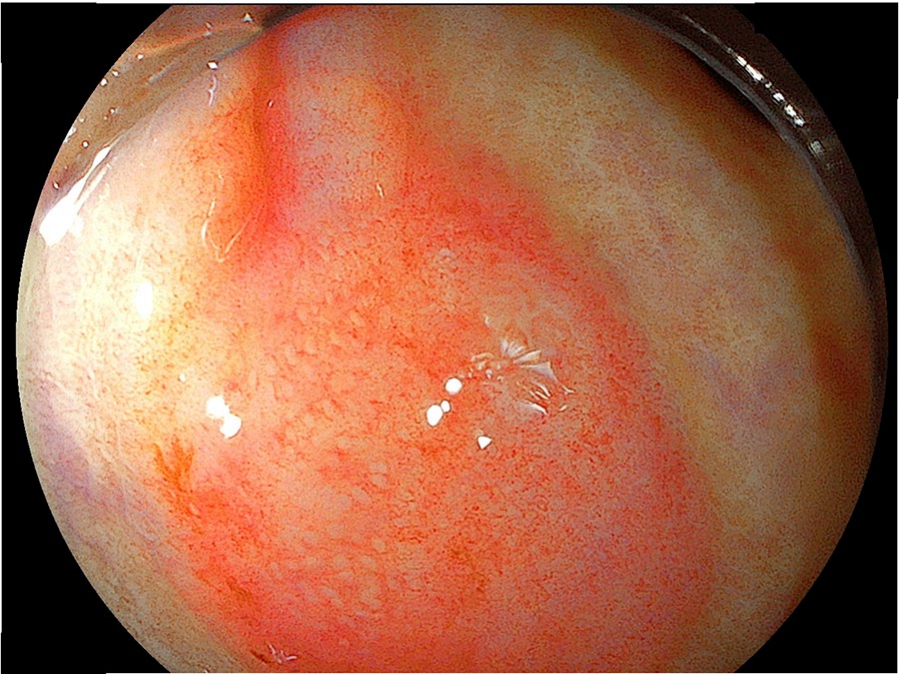
Endoscopic manifestations in Case 1. An elevated lesion with redness is visible in the descending colon, and it was biopsied for diagnosis

**Fig. 3:**
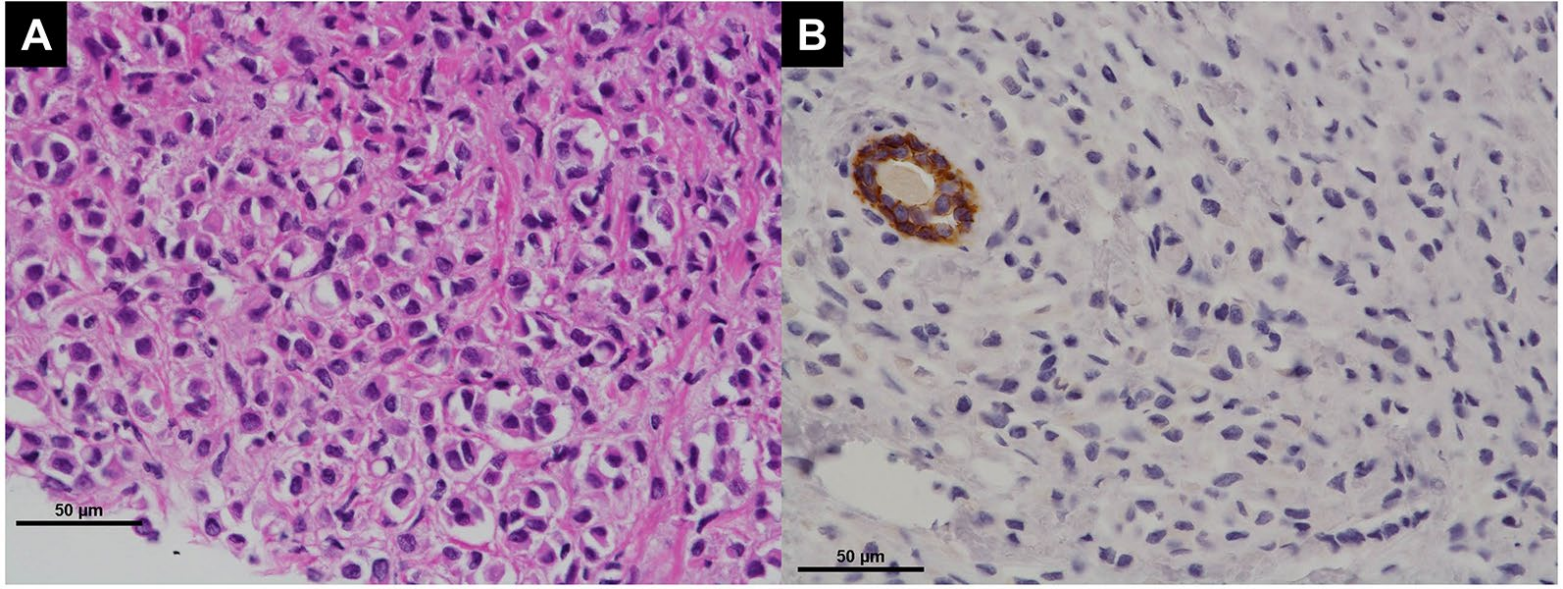
Histopathologic findings of breast cancer in Case 1 (invasive lobular carcinoma). **A** Atypical cells with large mucin vacuoles, which mimicked signet ring cell carcinoma, were seen (hematoxylin and eosin [HE]. Bar: 50 μm). **B** Tumor cells were negative for E-cadherin expression. (E-cadherin stain. Bar: 50 μm)

**Fig. 4:**
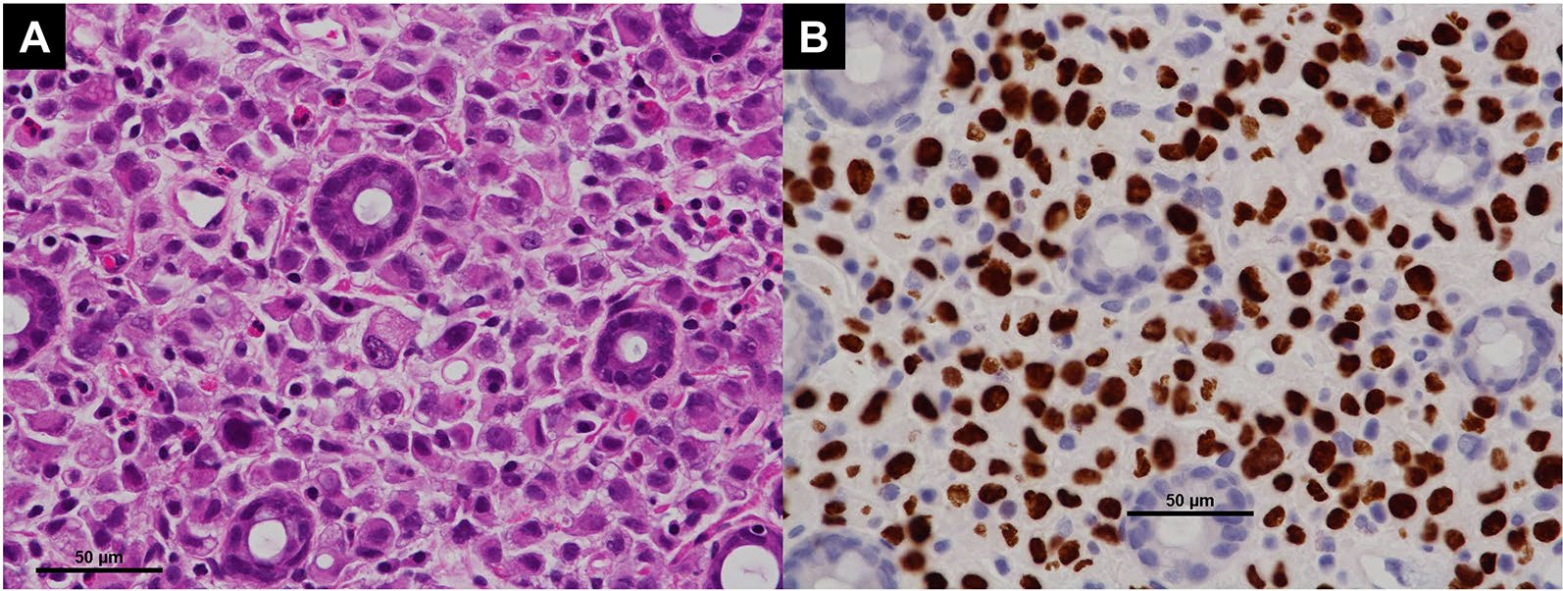
Histopathologic findings of colonic lesion in Case 1. **A** As with the breast lesion, tumor cells had clear droplets of mucin and nucleus displaced to the periphery. (HE stain. Bar: 50 μm). **B** GATA-3 positivity was demonstrated in tumor cells. (GATA-3 stain. Bar: 50 μm)

A repeat examination two days later revealed a torose lesion with redness in the descending colon, which was biopsied for diagnosis (Fig. 2). There was no evidence of tumor bleeding. Tumor marker tests indicated elevation of carcinoembryonic antigen (CEA) (43.7 ng/mL) and cancer antigen 15-3 (> 300 U/mL). Mammary gland ultrasonography revealed 19 mm and 16-mm-sized nodules in the left EAC and C segments, respectively. Histological examination of the left mammary nodule was negative for E-cadherin, leading to the diagnosis of ILC. The estrogen receptor (ER) and progesterone receptor (PgR) statuses were strongly positive, whereas HER2 was negative (Fig. 3). The colonic lesion had a histopathological appearance similar to that of signet ring cell carcinoma and was ER-positive, PgR-negative, and GATA-binding protein 3 (GATA3) -positive (Fig. 4). She was diagnosed with ILC with colonic and multiple bone metastases; however, considering her general condition, the best supportive care (BSC) was offered. The patient died 4 months after the diagnosis was confirmed.

### Case 2

**Fig. 5:**
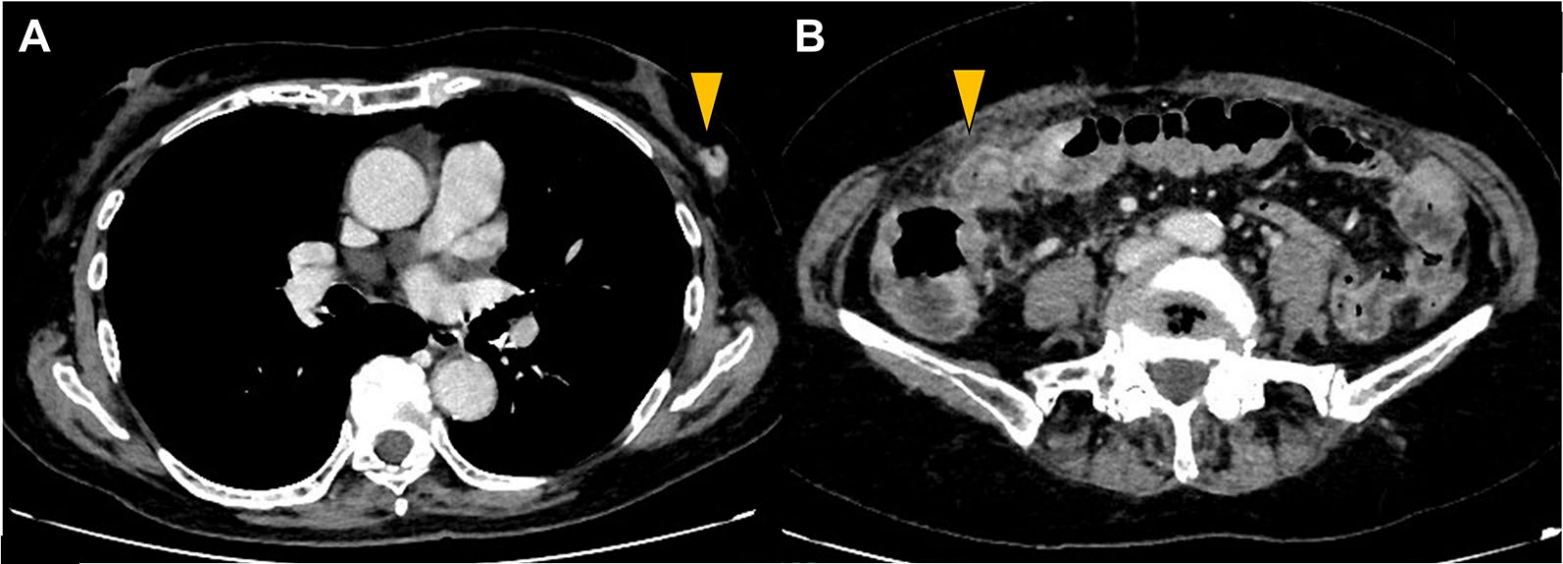
Computed tomography findings in Case 2. **A** A 14-mm-sized nodule is shown in the left mammary grand (arrowhead). **B** Bowel wall thickening is visible on the right side of the transverse colon, and density of the omentum is increased (arrowhead)

**Fig. 6:**
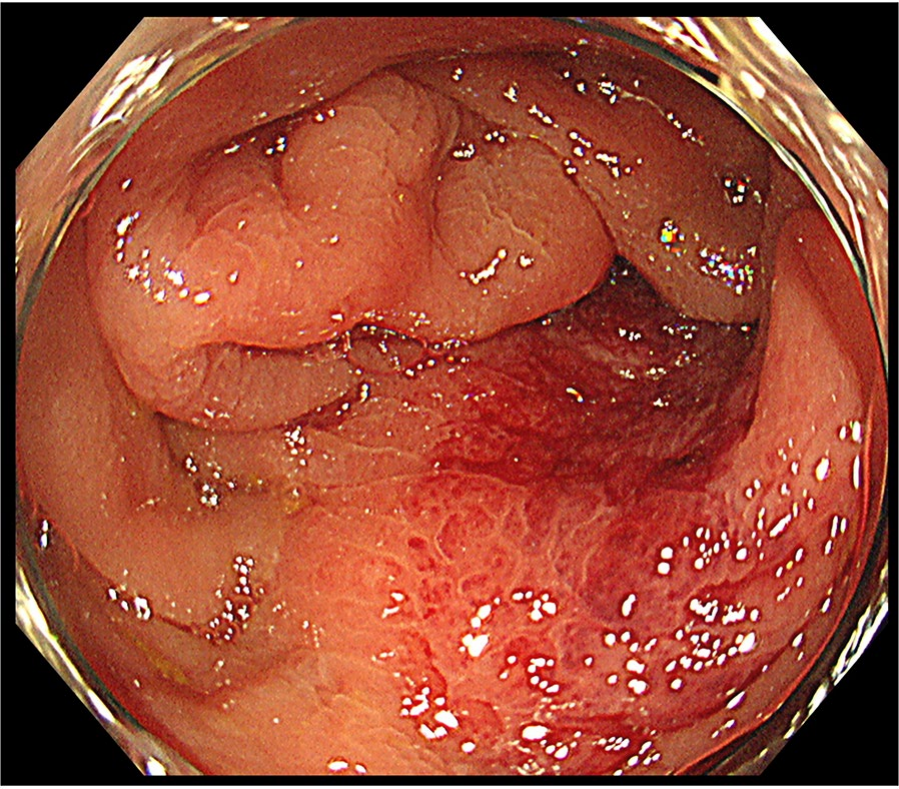
Colonoscopic findings in Case 2. The transverse colon is visibly narrowed by tumor spread

An 80-year-old female patient presented with diarrhea and vomiting. A CT scan suggested left breast cancer with ascending to transverse colon metastases and peritoneal dissemination (Fig. 5). Ultrasonography revealed a 14-mm nodule in the left C segment, which was diagnosed as ILC, characterized by positive ER and PgR expression and negative HER2 expression. A colonoscopy revealed stenosis of the right side of the transverse colon with tumor spread (Fig. 6). The histopathological diagnosis was ILC metastases, with tumor cells positive for ER and GATA3 and negative for E-cadherin. Ileosigmoid bypass surgery was performed for intestinal obstruction, and anastrozole was initiated on postoperative day six. After 2 months, the treatment for breast cancer was changed to fulvestrant and palbociclib because the peritoneal metastases had enlarged. Eight months after the operation, the tumor metastasized to the bone, and eribulin treatment was initiated. However, the patient developed peritoneal carcinomatosis and died 1 year and 2 months after the intestinal bypass.

### Case 3

A 56-year-old female patient underwent a left total mastectomy and axillary dissection for ILC that was ER-positive, PgR-positive, HER2-negative, and gross cystic disease fluid protein (GCDFP) 15-positive. Bone metastasis was shown six years later despite postoperative hormonal therapy; the treatment was switched to toremifene and zoledronic acid, and the tumor was under control. Nine years and 2 months after the breast surgery, she had an elevated CEA level (8.5 ng/ml) and a large tumor was detected in the transverse colon. Therefore, laparoscopic transverse colectomy was performed for the colonic lesion. Pathologically, it was similar to previous breast cancers in that small tumor cells with high nuclear-to-cytoplasmic ratio infiltrated, however, immunostaining for PgR and GCDFP 15 was negative, which was different from them. At that time, it was diagnosed with poorly differentiated adenocarcinoma of the colon, and postoperative chemotherapy with FOLFIRI (5-fluorouracil, folinic acid and irinotecan) was performed in accordance with advanced colon cancer.

**Fig. 7:**
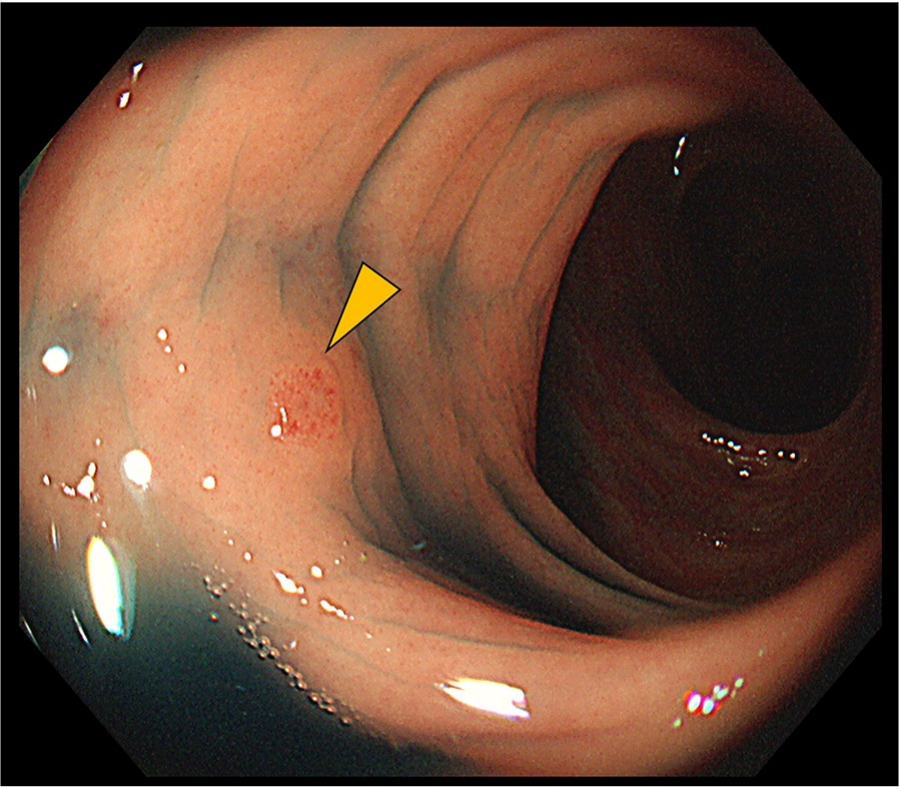
Endoscopic manifestations in Case 3. A torose lesion is shown in the descending colon (arrowhead)

**Fig. 8:**
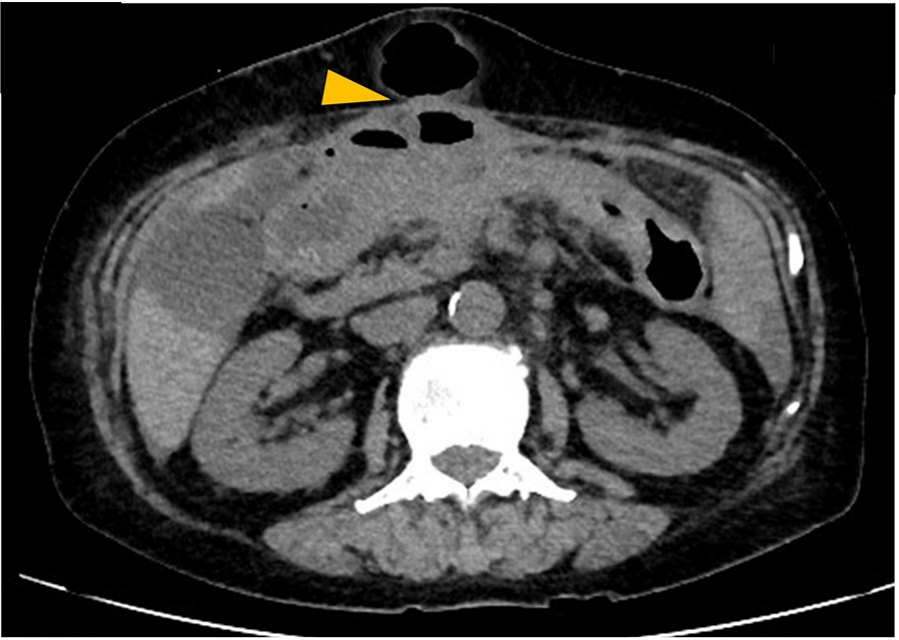
Abdominal computed tomography findings in Case 3. There is continuous subcutaneous air with transverse colon wall thickened by the tumor (arrowhead)

Two years and 2 months after the colectomy, torose lesions were detected in the hepatic flexure and descending colon (Fig. 7). The histological findings of these tumors mimicked those of previous transverse colon tumors and breast cancers, with positivity for ER and GATA3 and negativity for E-cadherin. All colon tumors, including previously resected tumors, were diagnosed as ILC metastases. Systemic treatment was continued; however, multiple metastases to the transverse colon, bilateral ovaries, and peritoneal metastases gradually progressed. Two years and 11 months after the abdominal surgery, the transverse colon lesion penetrated the abdominal wall, and an abscess formed. The patient underwent continuous suction drainage following ileostomy (Fig. 8). As the fistula improved 1 month later, vinorelbine treatment was started. Subsequently, colonic penetration recurred, and the patient died 3 years and 8 months after the colectomy.

## Discussion

The time interval between the initial breast cancer and gastrointestinal metastasis varies considerably from synchronous presentation to > 20 years after the diagnosis of the primary lesion [10, 11]. Mutations in E-cadherin, which contributes to cell adhesion, may be related to the susceptibility of ILCs to spread to the gastrointestinal tract [12, 13]. In approximately 1% of cases, gastrointestinal involvement is found to be the first distant metastasis [14]. For an accurate diagnosis, it is necessary to evaluate the histopathological characteristics of primary and metastatic foci. In terms of immunohistochemical surveys, ER and GATA3 positivity and caudal type homeobox 2 (CDX2) negativity may support the diagnosis of gastrointestinal metastases from breast cancer. Additionally, the usefulness of mammaglobin and GCDFP15 molecular markers has been reported in several studies [6, 15]. However, the properties of hormone receptors are not always consistent with those of the initial lesions [16]. In the present study, the expression of PgR in Cases 1 and 3 differed between the foci.

Since the clinical manifestations associated with gastrointestinal metastases are nonspecific, including abdominal pain, diarrhea, nausea, and sometimes asymptomatic, breast cancer is rarely detected based on these symptoms [17, 18]. In this report, vomiting and melena were observed in Case 1, while Case 2 experienced diarrhea and vomiting. In Case 1, bleeding from a Dieulafoy ulcer was indicated, but this was not evident from the metastatic mass in the descending colon. Colonic metastases from breast cancer have been reported to show diffuse intestinal wall thickening and ulcerated or nodular lesions, and endoscopic findings often resemble primary colon cancer or inflammatory bowel disease [19, 20]. Although a biopsy of the hemorrhagic site was not performed in this case, tumor infiltration may have played a role in the colorectal bleeding.

In Case 2, as metastatic masses in the transverse colon were responsible for intestinal obstruction, an ileosigmoid colon bypass was performed. Rapid examination and diagnosis of primary and metastatic lesions enabled early hormonal treatment after the improvement of abdominal manifestations.

Although Case 3 had no gastrointestinal symptoms, colonic metastasis was found due to elevated CEA levels during the postoperative follow-up of ILC. However, once the metastatic focus was resected, the tumor recurred and penetrated the abdominal wall. Colonic perforation or infiltration of the abdominal wall, which is implicated in metastatic lesions, is extremely rare. This was possibly associated with rapid tumor growth and increased bowel pressure due to intestinal stenosis. Also, high tumor invasiveness due to loss of E-cadherin might be related to this phenomenon. In this case, the fistula gradually shrank with surgical drainage, and temporary tumor regression was observed with chemotherapy. It was an intractable disease, and the metastatic lesion re-enlarged, causing a recurrence of the abdominal wall perforation and an abscess 8 months later. Nevertheless, the surgical treatment for gastrointestinal manifestations followed by systemic therapy for primary breast cancer was effective similar to that in Case 2.

Survival after gastrointestinal metastases is generally poor, and few patients survive for more than 2 years [6]. In this report, Case 1, in which BSC was provided, died 4 months after diagnosis. In contrast, the survival time was 1 year and 2 months after the detection of gastrointestinal metastasis in Case 2 and 3 years and 8 months in Case 3, who underwent systemic treatment subsequent to invasive procedures.

## Conclusions

When colonic foci are suspected in patients with breast cancer, especially ILC, the possibility of distant metastasis should be considered despite its rare nature. Metastasis to the gastrointestinal tract can trigger a wide range of abdominal symptoms and sometimes contribute to the detection of the primary lesion. Since the prognosis for these cases is poor, prompt examination of both foci is essential. Furthermore, in select cases, it is reasonable to consider surgical intervention for gastrointestinal metastasis to alleviate abdominal symptoms and lead to systemic therapy.

## Data Availability

Not applicable.

## Abbreviations

BSC: Best supportive care
CEA: Carcinoembryonic antigen
CT: Computed tomography
ER: Estrogen receptor
PgR: Progesterone receptor
GATA3: GATA-binding protein 3
GCDFP15: Gross cystic disease fluid protein 15
CDX2: Caudal type homeobox 2
